# Learners' decisions for attending Pediatric Grand Rounds: a qualitative and quantitative study

**DOI:** 10.1186/1472-6920-6-26

**Published:** 2006-04-27

**Authors:** Jack L Dolcourt, Grace Zuckerman, Keith Warner

**Affiliations:** 1 Division of Neonatology, Department of Pediatrics, University of Utah School of Medicine, and Primary Children's Medical Center, Salt Lake City, Utah, USA; 2 School of Education, Westminster College, Salt Lake City, Utah, USA; 3 Student, University of Utah, Salt Lake City, Utah, USA

## Abstract

**Background:**

Although grand rounds plays a major educational role at academic medical centers, there has been little investigation into the factors influencing the learners' decision to attend. Greater awareness of attendees' expectations may allow grand rounds planners to better accommodate the learners' perspective, potentially making continuing education activities more attractive and inviting.

**Methods:**

We used both qualitative (part A) and quantitative (part B) techniques to investigate the motivators and barriers to grand rounds attendance. Part A investigated contextual factors influencing attendance as expressed through attendee interviews. Transcripts of the interviews were analyzed using grounded theory techniques. We created a concept map linking key factors and their relationships. In part B we quantified the motivators and barriers identified during the initial interviews through a survey of the grand rounds audience.

**Results:**

Sixteen persons voluntarily took part in the qualitative study (part A) by participating in one of seven group interview sessions. Of the 14 themes that emerged from these sessions, the most frequent factors motivating attendance involved competent practice and the need to know. All sessions discussed intellectual stimulation, social interaction, time constraints and convenience, licensure, content and format, and absence of cost for attending sessions. The 59 respondents to the survey (part B) identified clinically-useful topics (85%), continuing education credit (46%), cutting-edge research (27%), networking (22%), and refreshments (8%) as motivators and non-relevant topics (44%) and too busy to attend (56%) as barriers.

**Conclusion:**

Greater understanding of the consumers' perspective can allow planners to tailor the style, content, and logistics to make grand rounds more attractive and inviting.

## Background

In William Ostler's era of the early 1900s, grand rounds was a bedside teaching tour led by master clinicians, and subsequently it has evolved into a lecture series[1,2]. Grand rounds' styles reflect institutional tradition, and its themes and content reflect the perspective of the clinical departments[3]. Competing time demands, convenience, relevance of topics, and speaker skills all can influence the decision whether or not potential attendees allocate time for grand rounds[4]. Concerns about the ongoing relevance of grand rounds[5] as reflected in attendance patterns have been studied in the United States, Canada, Australia, and New Zealand, and inferences have been made based on provider data or perceptions [6-12]. We wanted to explore, from the learner's perspective, the factors influencing the decision for attending grand rounds and compare those with the provider-identified objectives for grand rounds.

A large fraction of the potential grand rounds audience chooses not to attend. According to one survey, nearly half of full-time and two-thirds of part-time department of medicine faculty missed more than half of grand rounds sessions[10], and more than 40 percent of departmental representatives perceived the popularity and attendance were decreasing[7]. Successful attendance-building strategies include improving the understanding of attendance patterns, educational needs and preferences, the scheduling of notable or timely sessions, increasing attendance by residents and researchers, and increasing publicity[9]. One proposed attendance-building strategy is to apply adult learning principles, which includes selecting meaningful and relevant topics[11]. Greater knowledge of the details of the audience's decision-making process could reveal (1) more specific guidelines for what constitutes meaningful and relevant topics, (2) interrelationships between decision factors, and (3) the relative importance of specific decision-making factors.

### Purpose of grand rounds

Grand rounds are expensive to produce, attract a large audience and providers perceive them to be of high quality[6-8,10]. They can be an effective vehicle for information transfer, though sometimes the information disseminated may be conflicting or erroneous[12]. Grand rounds as an educational event has been investigated primarily through multi-institutional surveys using questionnaires sent to planners, departmental chairs, and residency directors in the United States[7], Canada[6,8], and Australia[11]. According to these surveys, the purpose of grand rounds, from the providers' perspective, includes presenting research findings by departmental scholars, showcasing "stars" of the physician staff, updating diagnosis and treatment, educating faculty and house staff, and social interaction[6,7]. We found no similar description of goals reflecting the audience's viewpoint. Furthermore, assessment of learners' needs and speaker evaluation may be inconsistently used for planning purposes[8,10]. In the business model, success depends on satisfying customer needs, and marketing concepts reflect an understanding of the behaviors influencing purchasing decisions[13]. In healthcare education where participation is voluntary and success depends on satisfying learner needs, a better understanding of the learners' considerations is a potential tool for increasing attendance.

### Purpose of this research project

We used both qualitative and quantitative investigative techniques to identify and prioritize motivating factors and barriers for attendance. For part A of this study, we used interviews to qualitatively investigate the context of grand rounds in professional development. Qualitative research is better suited than quantitative methods for revealing the factors in the decision-making process itself because (1) the experience may vary among individuals and should be captured in their own words, (2) the process is fluid and dynamic and cannot be fully summarized on a single rating scale, and (3) the participants' perceptions are key to the process[14]. We applied the grounded theory approach, which allows the theory to emerge from the data[15] by using interviews to uncover (1) the factors affecting attendees' decisions to attend Pediatric Grand Rounds presentations, (2) the perceived desirable content and instructional characteristics for Pediatric Grand Rounds, and (3) how Pediatric Grand Rounds fits into the attendees' overall scheme of continuing professional development. Because qualitative analysis identifies factors but is unable to assign a relative weight, in part B we used an audience survey to quantitatively measure the relative importance of the motivating factors and barriers that had emerged during the first two interview sessions.

## Methods

The Pediatric Grand Rounds target audience consists of practicing academic medical center and community physicians, including general and specialty pediatricians, advanced practice nurses, residents, non-physician professionals, and medical students at a not-for-profit pediatric specialty hospital that is independently-owned but has extensive ties to the state university medical school. Of the 506 persons who attended one or more Pediatric Grand Rounds sessions during the period January 1 through December 31, 2002, 299 (59%) were physicians and 207 (41%) were non-physicians. In part A of this study, four groups of potential study subjects were recruited based upon actual attendance. Group 1 consisted of all 13 known retired physicians. Group 2 consisted of the 40 most-frequent academic medical center-based physician attendees and community-based physician attendees. Group 3 consisted of the 20 most frequent non-physician attendees. Group 4 consisted of 40 physicians who attended infrequently (2–5 sessions). Potential study subjects were sent personal letters inviting them to participate in a one-time group interview. We conducted a total of 7 one hour group interviews arranged to accommodate the participants' schedules. Each group consisted of 1–3 study subjects seated around a table or convened via conference call, while one or two of the authors (JD and GZ) acted as moderators. Participation was voluntary, and there were no inducements. Each interview group was asked to identify (1) the reasons for attending Pediatric Grand Rounds, (2) desirable features in Pediatric Grand Rounds, (3) factors motivating attendance, and (4) how Pediatric Grand Rounds fits into their personal continuing education and professional development. The group interview research project was approved by the University of Utah Institutional Review Board.

The session transcript was distributed to each of the participants and corrected for accuracy and completeness. Sessions were concluded after the 7^th ^group interview because no new themes had emerged. Line-by-line analysis was carried out to generate categories and relationships among categories and to discover their characteristics[10]. A concept map was distributed to each of the participants to verify accuracy and completeness.

The quantitative survey (part B) consisted of 2 multiple choice, multiple response questions that was part of an annual anonymous satisfaction questionnaire widely distributed by mail to 442 persons routinely receiving the monthly grand rounds calendar and was also available for pick-up on-site prior to each grand rounds session for 4 consecutive weeks during Spring 2002. With the exception these two study questions, the content and format of the annual questionnaire is unchanged from year-to-year. The response choices for the question, "What motivates you to attend grand rounds?" were continuing education credits needed for license, networking with colleagues, clinically/practically useful topics, cutting edge research, and refreshments. The response choices for the question, "What prevents you from attending grand rounds?" were topics not relevant to my practice, regularly scheduled meeting conflicts, busy schedule prevents me from getting away and child care issues. The response choices for the respondent's profession were academic/university pediatrician, community pediatrician, family practice physician, retired physician, fellow, resident, nurse practitioner, staff nurse, pharmacist, physician's assistant, student and other. All questions allowed additional free-form responses. These questions were not pilot tested prior to inclusion on the annual survey. There were no inducements for returning a completed survey.

## Results

### Part A: interviews

Sixteen persons [14 pediatricians (5 retired community generalists, 1 semi-retired pediatric specialist, 2 faculty specialists, 4 faculty generalists, and 2 community generalists) and 2 administrative pediatric nurses] took part in one of seven group interviews. All held active professional licenses. No one in the infrequent attendance group (physicians who attended 2–5 sessions) participated in any interview. All quotations are referenced by interview session number (I#).

Fourteen themes emerged from the interviews: competency, professional development, intellectual stimulation, consultation, referral, social interaction, licensure, instructional content and format, time constraints and convenience, lack of cost, availability of other educational venues, malpractice insurance, providing voluntary medical services, and retirement. The educationally-related themes were lumped into 6 categories: licensure, competent practice, social, entertainment, convenience factors, and rationale for attending grand rounds. Many of the themes were common to all sessions. Competent practice was not cited by retired pediatricians, but all other categories were identified by all groups, with varying degrees of emphasis. All groups utilized other resources beyond grand rounds for continuing professional education, usually attending local and national courses and reading journals. Retired physicians were especially drawn to grand rounds because it provided no-cost continuing medical education (CME) credit necessary for meeting state licensure requirements. As one physician explained:

"But I usually manage to attend all of them for the CME credit, which aids me in renewing my license...As long as I can enjoy good health, I will enjoy rounds...It is cost effective, it's convenient, and it's one I can get to easily...So I think that that's an issue also when you retire when you have to budget a little more carefully." (I1)

### Content

General and specialist pediatricians agreed that presentations should emphasize general pediatric topics that include practical points and the latest science. One community-based pediatrician stated:

"It's nice to see a variety in Grand Rounds; that is to say, it's not always practical pediatrics, it's not always evidence-based medicine, and it varies a lot...As far as features that I look for, I'm inclined to go for a variety rather than one consistent theme. I think the Grand Rounds that are the more interesting may not be the most practical. The ones that are the most interesting are the ones that provoke thought and which also sometimes provoke controversy. So I don't think there should be one overriding motive or one overriding feature to look for in Grand Rounds." (I5)

Generalists felt that the content should never reach the complexity of specialist care. An academic medical center-based pediatrician explained:

"I think it is important to keep a focus of interest to the general academic pediatric community and etc. The subspecialists, if they are so inclined, can go to their rounds, they probably have Neurology Grand Rounds, or Surgical Grand Rounds, Ortho Grand Rounds, etc., so that keeping it focused on the pediatric aspect of generalists is more important." (I5)

In contrast, the specialists did not mention the need for practical applications, and felt that their goal was to maintain familiarity with a wide range of general pediatric topics. One person stated:

"What pediatric grand rounds allows me to do as a pediatric subspecialist is to maintain that pediatric part as opposed to the neonatology part. Obviously, there is a fair amount of overlap but, that's most of the Pediatric Grand Rounds, and appropriately so, you know, are focused upon issues that are more part of the day by day needs of pediatricians rather than subspecialists and just allows me to kind of keep that going and keep perspective, I think, in some ways so that I can get a feel for how pediatrics that I learned so many years ago has continued to grow and develop in areas other than what I practice on a day by day basis so I find it important for that. I think it is something that we tend to neglect when we are, you know, busy all the time. I think it is an important part of being a pediatric subspecialist, that is, maintaining a perspective of what pediatricians are doing and what is of interest to them and so Pediatric Grand Rounds is really a very efficient way of allowing me to do that." (I4)

Whether practicing in the community or at the academic medical center, pediatricians did not subscribe to a dichotomous choice between scientific research and clinical medicine, and felt that both aspects should be represented in grand rounds presentations. One community pediatrician explained:

"You know, I would say it still needs to be mixed. I think, even though, certainly a generalist's goal, a large percentage of attendees...are from the faculty and I think you need to treat those two audiences at the same time and so, I do think that there needs to be some new science, not that the generalists wouldn't be interested in new science, but that I wouldn't only direct the content at, you know, what someone in private practice, for example, could use tomorrow. I think it needs to be a mix of understanding the latest science." (I6)

### Entertainment

Grand rounds has entertainment value. As one pediatrician stated:

"...it's like a magazine show, you know, TV magazine, entertainment to me, I don't really understand them, but it is entertainment again. It kind of opens up my eyes that there is the knowledge explosion that is going on. My gosh, it's overwhelming." (I2)

Local faculty may be perceived as luminaries, as one pediatrician observed:

"Yes, it's fun to hear the big guns when you go out of town to a meeting, but there are big guns right here in our own community..." (I1)

Respondents valued presentation skills such as the presenter's dynamism, humor, clear and concise presentations, and explained the value of handouts:

"First of all, you know that it will be concise, it will be well presented...Their handouts are usually good for later use if I miss something along the way." (I1)

Panel discussions and presentations with multiple presenters were perceived as unattractive because they often lacked unity, or were not perceived as having sufficient depth.

### Situational factors

Some factors were uniquely important to certain groups. For retired physicians, the most important factors for attending were maintaining professional identity, the need for no-cost CME credit, and favorable weather conditions. Practicing pediatricians uniquely identified the need for current knowledge to teach residents. Retired and community pediatricians were the only groups to identify the importance of convenient parking.

Free CME credit was an important motivator for all physician groups, but was mentioned most frequently by the group of retired physicians living on a fixed income. In contrast, continuing education credits were not important to the nurses because credits are not required for renewal of the state nursing license. All four professional groups (retired physicians, academic physicians, community physicians, and administrative nurses) use grand rounds as a venue for networking: professional relationships are maintained or renewed, messages to colleagues are related, brief consultations are carried out, and sub-specialists are identified for patient referral.

**Figure 1:**
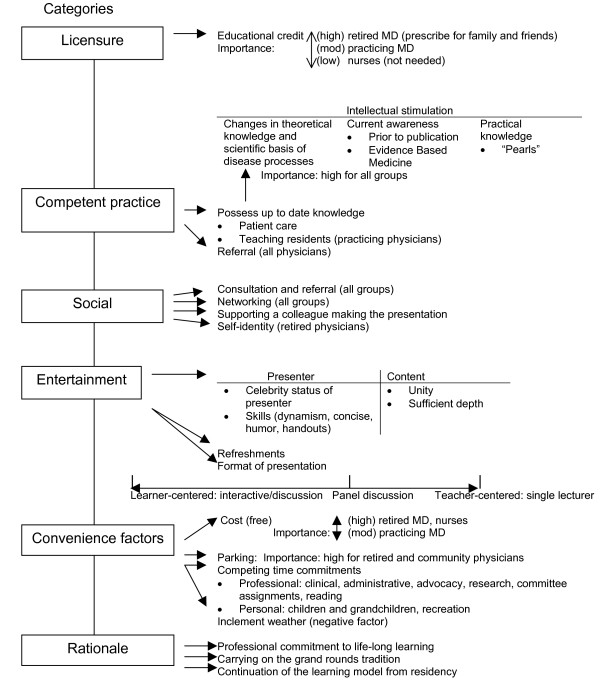
Concept map of factors affecting grand rounds attendance.

Based on grounded theory analysis, we constructed a concept map (Figure 1) demonstrating the relationships among factors affecting grand rounds attendance.

### Part B: audience survey

Fifty-nine questionnaires (11.7% of those attending 1 or more session) were returned and categorized into 3 groups. Group 1 (academic medical center practice) consisted of academic medical center pediatricians and advanced practice nurses. Group 2 (community practice) consisted of community pediatricians, family practice physicians, and retired physicians. Group 3 (others) consisted of students, residents, fellows, physician assistants, staff nurses, and other hospital staff. Forty-two (71%) of the questionnaires had been picked up on-site. The results are demonstrated in Table 1.

## Discussion

Ours is the first study to characterize motivations for attending grand rounds from the learners' – rather than from the planner's – viewpoint. The combination of qualitative (part A) and quantitative (part B) data allowed us to identify key factors and their relationships, as well as prioritize their relative importance. Clinical utility of the topic, being a competent practitioner, intellectual stimulation, social interaction, and entertainment were the most important motivators. Sometimes the boundaries were indistinct. This overlap can be explained by the inherently scientific nature of professional practice ingrained in physicians at all levels of training, from medical school through residency and fellowship. Achievement, curiosity, professionalism, CME credit, social interaction, and enjoyment are known motivators for self-directed learning[16,17]. By its inherent design, this study emphasized the internal motivating factors, though the second most important factor was the external motivator of CME credit.

All physicians, whether retired or in active practice, consistently cited keeping up-to-date as an integral component of professionalism. Up-to-date knowledge is a necessary, but not sufficient, condition for competence. This distinction was best exemplified by a group of retired physicians who expressed their desire to remain informed, but at this point in their careers, none of them claimed to be competent practitioners. Competency as a motivating force is consistent with Putnam and Campbell's view that that a physician's desire for competence is manifest in (1) striving to excel and be the "best" in the field, (2) willingness to change and innovate in clinical practice, and (3) willingness to respond to clinical problems[18]. Cutting-edge research was a modest motivator for all groups, especially for those practicing outside of the academic medical center. All these factors contribute to the assessment of a topic's relevance. When the attendee judges the topic irrelevant, this negative perception becomes the second most important attendance barrier. The questionnaires, but not the interviews, identified scheduling conflicts as the greatest barrier for all groups. We speculate that selection bias best explains why scheduling conflicts were never identified during the interviews because frequent attendees had been generally successful in resolving such conflicts.

Previously unidentified factors motivating learners to attend grand rounds are (1) striving for competent practice, (2) retired physicians maintaining their self-identity, (3) garnering non-cost continuing medical education credits, and (4) being entertained (beyond the perception of being an engaging speaker). The concurrence between the results of our study of learners vs. providers demonstrates that learners and administrative planners agree that grand rounds should (1) provide updates in diagnosis and treatment, (2) provide updates in medical research, (3) educate housestaff and faculty, and (4) facilitate clinical referrals[7]. Both the administrative planners and learners recognize the role of grand rounds as a social event. Medicine department heads recognize the importance of grand rounds for promoting a collegial atmosphere which contributes to the sociological concept of "bridging capital" – the ability to transcend professional boundaries[19]. Similarly, the subjects in our study used the grand rounds environment for networking opportunities (i.e. obtaining informal medical consultations, communicating messages to colleagues, and renewing social acquaintances).

There are several limitations to this and previous studies. Previous reports based on multi-institutional questionnaires are unable to detect the more subtle factors which are obscured by audience heterogeneity[6-8,10,11]. A strength of our study is the homogeneity of our study population, allowing an in-depth probing of the decision-making process. However, because we studied only a single grand rounds series conducted at a single institution, our findings may not be universal and will need to be verified in other venues and specialty areas. This study's greatest limitation is the small number of volunteers for the interviews and respondents to the questionnaire. Since all volunteers for the interview were frequent attendees, the concept map may not adequately represent infrequent or non-attendees. The low response rate for the survey can result in an ascertainment bias. Furthermore, since the questionnaires were anonymous, we had no way to determine if respondents submitted multiple surveys. All of these factors could limit the validity of generalizing our findings to other audiences. Additional research is needed to determine if the factors we identified and the interrelationships outlined by the concept map are applicable to other types of audiences, other medical specialties, and other geographical regions.

## Conclusion

Despite their differing professional roles, all groups of grand rounds attendees valued clinically-useful topics, and identified intellectual stimulation, competence, continuing education credit for licensure, social interaction, entertainment value, and convenience as important motivating factors, though emphasis differed among the groups. Competing time demands, non-relevant topics, and inconveniences such as parking and inclement weather were the major negative motivating factors. Because of our research, we now ask presenters to design their grand rounds presentations so that the instructional content contains a mixture of current science and practical application and targeted to the level of the generalist.

As consumers of educational products, busy healthcare providers make choices among competing alternatives for their time. By recognizing these key decision factors, planners can potentially increase grand rounds attendance by structuring the style, content, and logistics to better accommodate the learners' perspective.

## Competing interests

The author(s) declare that they have no competing interests.

## Authors' contributions

JLD conceived and designed the study, obtained IRB approval, developed the interview and survey questions, recruited participants, participated in the subject interviews, coded transcripts, and drafted the manuscript. GZ participated in the design of the study, participated in the subject interviews and helped to draft the manuscript. KW participated in the subject interviews and helped to draft the manuscript. All authors read and approved the final manuscript.

## Pre-publication history

The pre-publication history for this paper can be accessed here:



## Figures and Tables

**Table 1:** Audience questionnaire results of motivators and barriers to attending Pediatric Grand Rounds

	Group 1^‡ ^n = 29	Group 2^‡ ^n = 17	Group 3^‡ ^n = 14	Total n = 59
Motivators*				
				
Clinically-useful topics	23 (79)	17 (100)	10 (71)	50 (85)
Continuing education credit	14 (48)	10 (59)	3 (21)	27 (46)
Cutting-edge research	6 (21)	7 (41)	3 (21)	16 (27)
Networking	5 (17)	6 (35)	2 (14)	13 (22)
Refreshments	1 (3)	2 (12)	2 (14)	5 (8)
Other	3 (10)	1 (6)	3 (21)	7 (12)
Barriers*				
				
Busy schedule	13 (45)	14 (82)	6 (43)	33 (56)
Non-relevant topic	13 (45)	9 (53)	4 (29)	26 (44)
Child care	4 (14)	0	0	4 (7)
Regular schedule conflict	3 (10)	0	0	3 (5)
Other barrier	3 (10)	0	0	3 (5)

( ) percent of respondents *all NS between groups by X^2^

^‡ ^Group 1: academic medical center practice

Group 2: community practice

Group 3: all others
